# Antecubital vein approach of retrograde transvenous obliteration using a steerable triaxial system for portosystemic encephalopathy

**DOI:** 10.1016/j.radcr.2023.06.040

**Published:** 2023-07-07

**Authors:** Fumio Chikamori, Satoshi Ito, Ryo Hamada, Niranjan Sharma

**Affiliations:** a Department of Surgery, Japanese Red Cross Kochi Hospital, 1-4-63-11 Hadaminamimachi, Kochi, 780-8562 Japan; b Department of Radiology, Japanese Red Cross Kochi Hospital, Kochi, Japan; c Adv Train Gastroint & Organ Transp Surgery, New Zealand

**Keywords:** Steerable triaxial system, Steerable microcatheter, Portosystemic encephalopathy, Antecubital vein, Portosystemic shunt, Retrograde transvenous obliteration

## Abstract

We report a case of portosystemic encephalopathy treated by retrograde transvenous obliteration (RTO) with an antecubital vein approach using a steerable triaxial system. A 77-year-old female was referred to our department complaining of dizziness and tremor. Laboratory data showed hyperammonemia. Contrast-enhanced CT and 3D-CT reconstruction images demonstrated an inferior mesenteric vein (IMV)-left common iliac vein shunt and a splenorenal shunt. The former was treated as a responsible shunt. The spleen volume was 212 mL, and the liver volume was 757 mL; giving a spleen/liver volume ratio of 0.3. Partial splenic artery embolization (PSE) was employed to control portal venous pressure. The hepatic venous pressure gradient (HVPG) changed from 13.2 to 9.6 mm Hg and the spleen/liver volume ratio improved from 0.3 to 0.2 by PSE. Two months after PSE, RTO with an antecubital vein approach using a steerable triaxial system was performed. HVPG changed to 12.5 mm Hg after RTO. Contrast-enhanced CT and 3D-CT reconstruction images 3 days after the procedure demonstrated the thrombus in the IMV-left common iliac vein shunt. We conclude that the antecubital vein approach using a steerable triaxial system is a feasible and minimally invasive technique in RTO for portosystemic shunts.

## Introduction

Hepatic encephalopathy secondary to portosystemic shunts [1,2] in patients without deteriorated liver function can be treated by shunt obliteration [3]. We have reported many techniques of shunt obliteration for portosystemic encephalopathies, such as trans-jugular retrograde obliteration [4,5], percutaneous trans-hepatic obliteration [6], endoscopic embolization [7], and percutaneous trans-retroperitoneal direct obliteration [8]. Of these procedures, retrograde transvenous obliteration (RTO) has become widespread in recent years as a treatment for portosystemic encephalopathy [9], [10], [11]. However, RTO approaches were only either trans-femoral or trans-jugular [9], [10], [11]. We report a new RTO approach and technique for portosystemic shunts; the Antecubital Vein approach for RTO using a steerable triaxial system [12], [13], [14].

## Case report

A 77-year- old female was brought to the emergency room with chief complaints of dizziness and tremor. She was diagnosed with primary biliary cholangitis 11 years ago and was being followed up by a local physician.

On admission, she had GradeⅡhepatic encephalopathy according to the West Haven criteria [15]. Laboratory studies revealed hemoglobin 11.3 g/dL [normal range, 13.5-17.4], total leukocyte count 4330/µL (3500-8000/µL), platelet count 16.4 × 10^4^ /µL (12.3 -33.1 × 10^4^ /µL), total bilirubin 1.4 mg/dL (0.3-1.3 mg/dL), albumin 3.8 g/dL (3.8-5.0 g/dL), aspartate transaminase 48 U/L (10-32 U/L), alanine transaminase 23 U/L (5-27 U/L), prothrombin time 74.5 % (70%-130%), Mac-2 binding protein glycosylated isomers 5.26 COI (2+) (<1.00), antimitochondrial antibody >400 U/mL (<7.0 U/mL), and antinuclear antibody >1:320 (<1:40). The plasma ammonia level was abnormally elevated to 193 µg/dL (12-66 µg/dL). The retention rate of indocyanine green at 15 minutes (ICG15) was 19.5 % (<10 %). Hepatitis B surface antigen and hepatitis C virus antibody were negative. The Child-Pugh score was 6 and the class was A. Magnetic resonance imaging of the brain revealed no abnormal findings. The symptoms improved with branched-chain amino acid administration, but the ammonia level remained high. So, she was referred to our department.

**Fig. 1:**
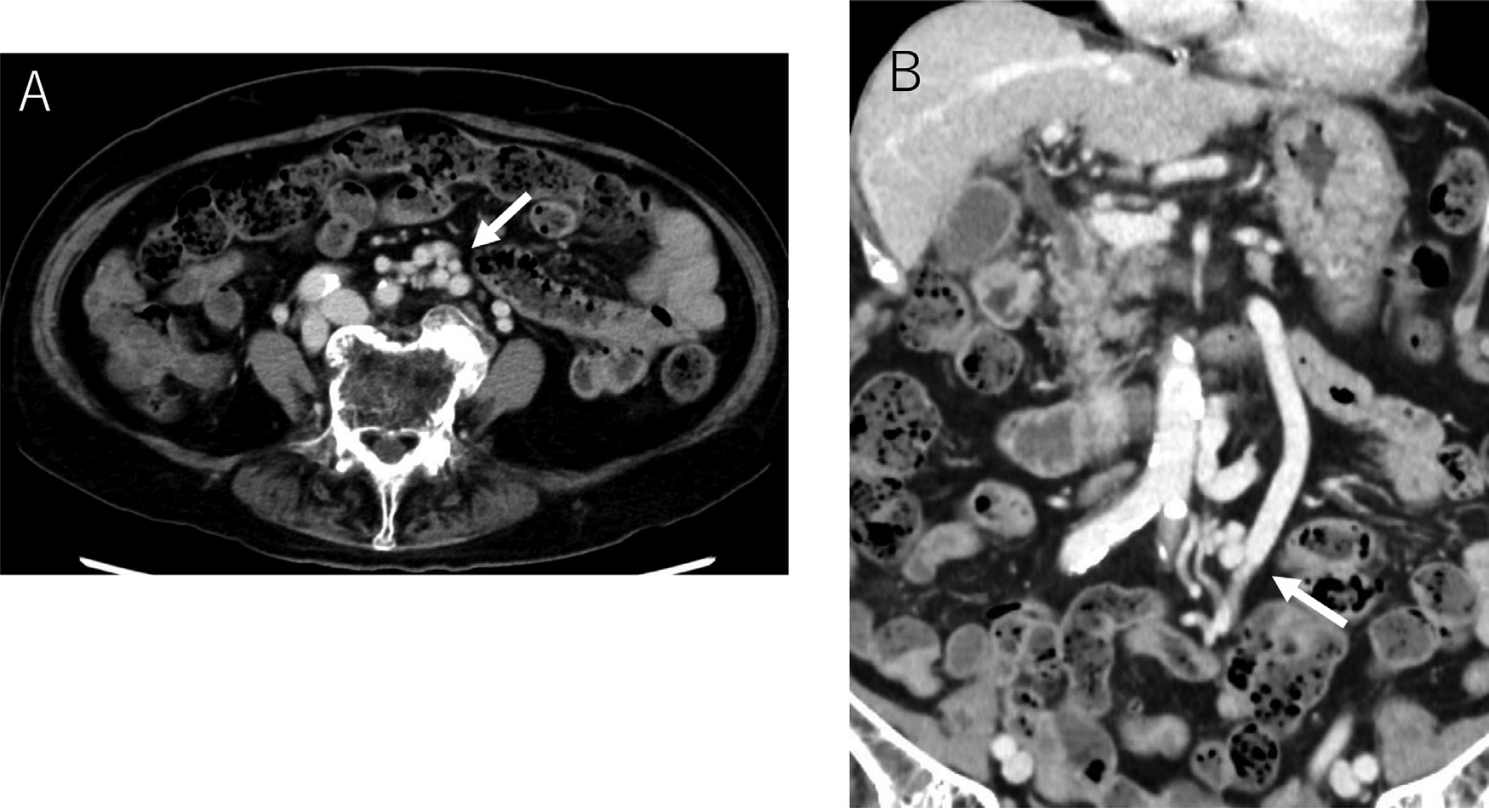
Contrast-enhanced CT before treatment. (A) Contrast-enhanced CT before treatment shows shunt vessels (white arrow). (B) Coronal view of contrast-enhanced CT before treatment shows dilated inferior mesenteric vein (IMV) (white arrow).

**Fig. 2:**
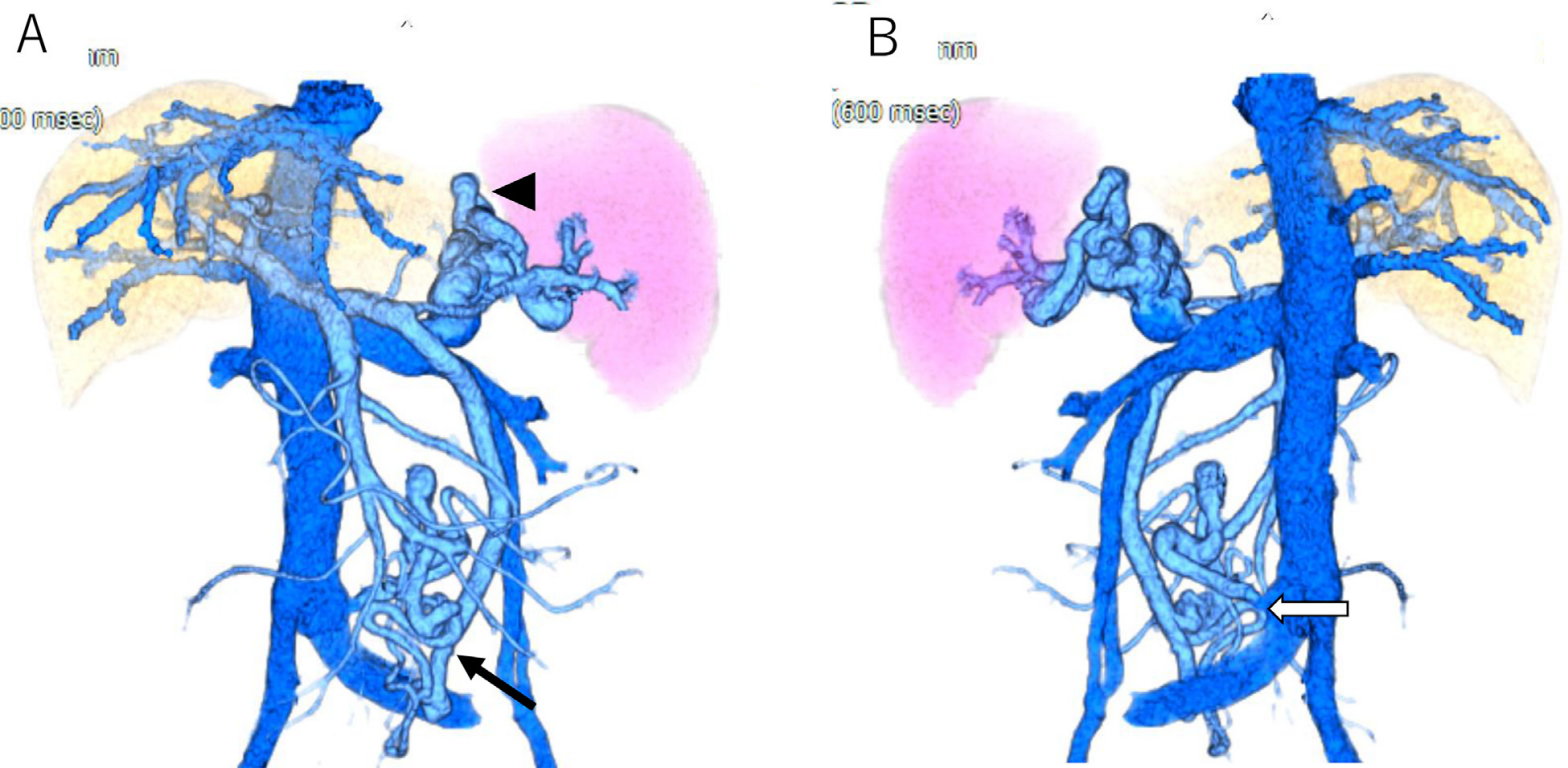
3D-CT reconstruction images before treatment. (A) Ventral view of 3D-CT reconstruction image before treatment shows IMV-left common iliac vein shunt (black arrow) and splenorenal shunt (black arrowhead). (B) Dorsal view of 3D-CT reconstruction image before treatment shows the confluence of the shunt to the iliac vein (white arrow).

**Fig. 3:**
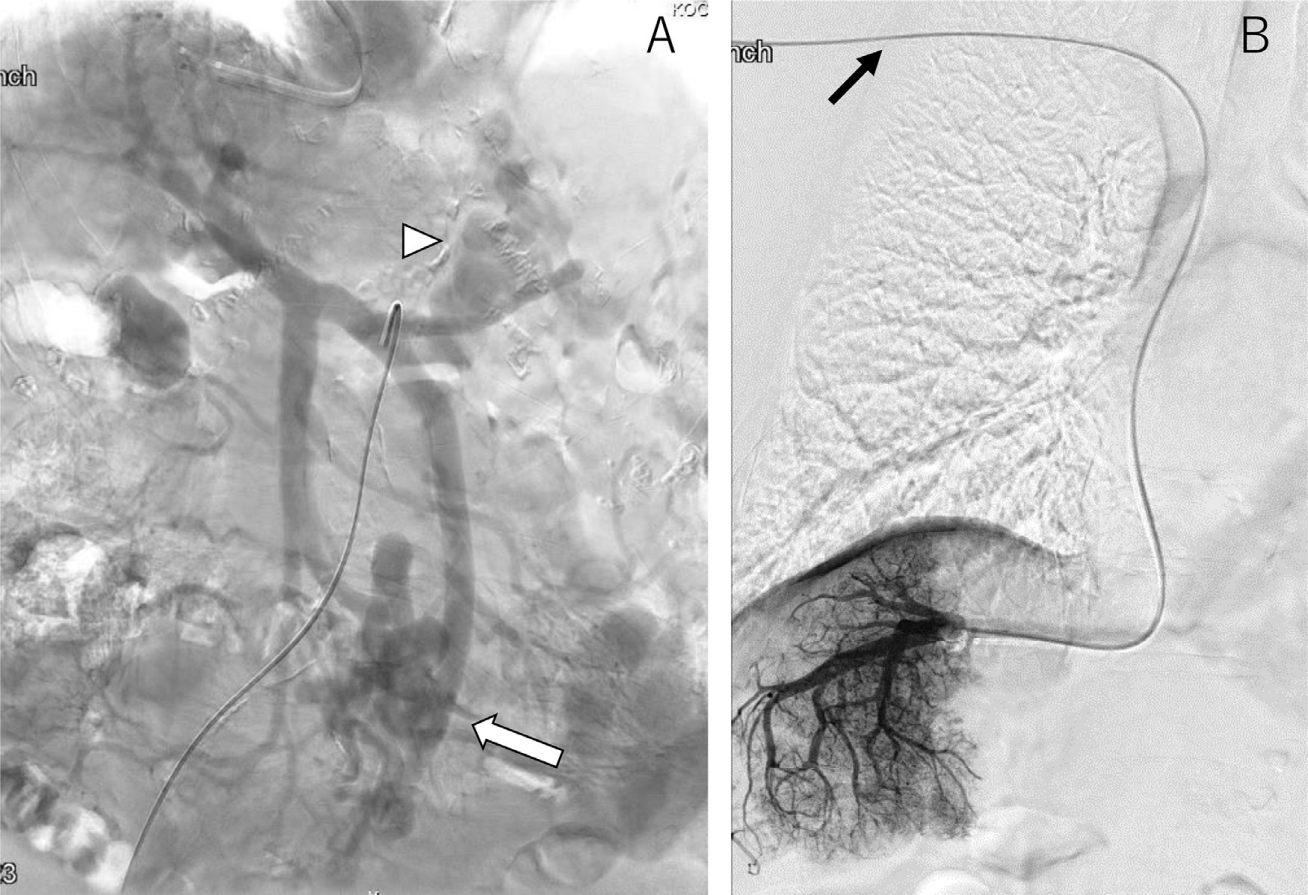
Portal phase of superior mesenteric arteriogram and retrograde hepatic venogram before RTO . (A) Portal phase of superior mesenteric arteriogram before RTO shows hepatofugal IMV-lt iliac vein shunt (white arrow) and splenorenal shunt (white arrowhead). (B) Retrograde hepatic venogram shows 5 Fr. balloon catheter (black arrow) is inserted via the antecubital vein.

**Fig. 4:**
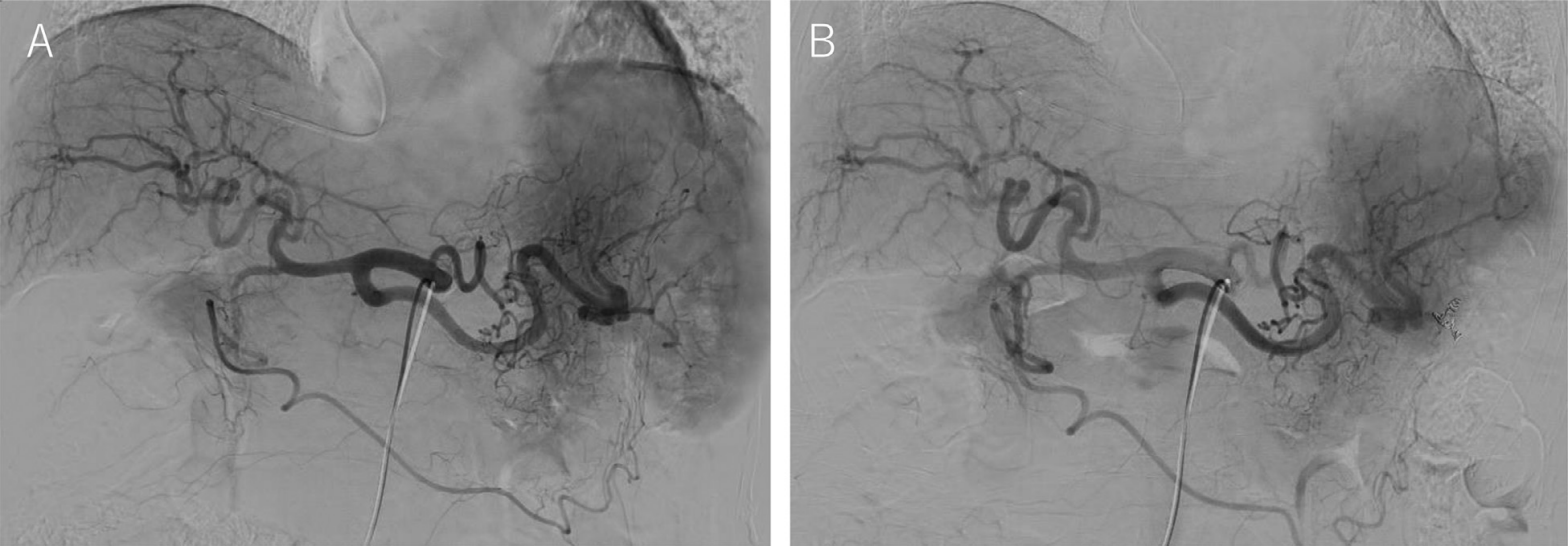
Celiac arteriogram before (A) and after (B) PSE.

Contrast-enhanced computed tomography (CT) and 3 dimensional (3D)-CT reconstruction images demonstrated an inferior mesenteric vein (IMV) - left common iliac vein shunt and a splenorenal shunt (Figs. 1A, B and 2A, B). The spleen volume was 212 mL, and the liver volume was 757 mL, giving a spleen/liver volume ratio of 0.3. The portal phase of superior mesenteric arteriography revealed the 2 hepatofugal portosystemic shunts: the IMV-left common iliac vein shunt and splenorenal shunt (Fig. 3A). As a policy of partial and stepwise shunts obliteration, we planned the former shunt obliteration. Prior to shunt obliteration, partial splenic artery embolization (PSE) was employed to control portal venous pressure. HVPG changed to 9.6 mm Hg from 13.2 mm Hg (Fig. 3B). The spleen/liver volume ratio improved to 0.2 after PSE (Fig. 4A and B).

**Fig. 5:**
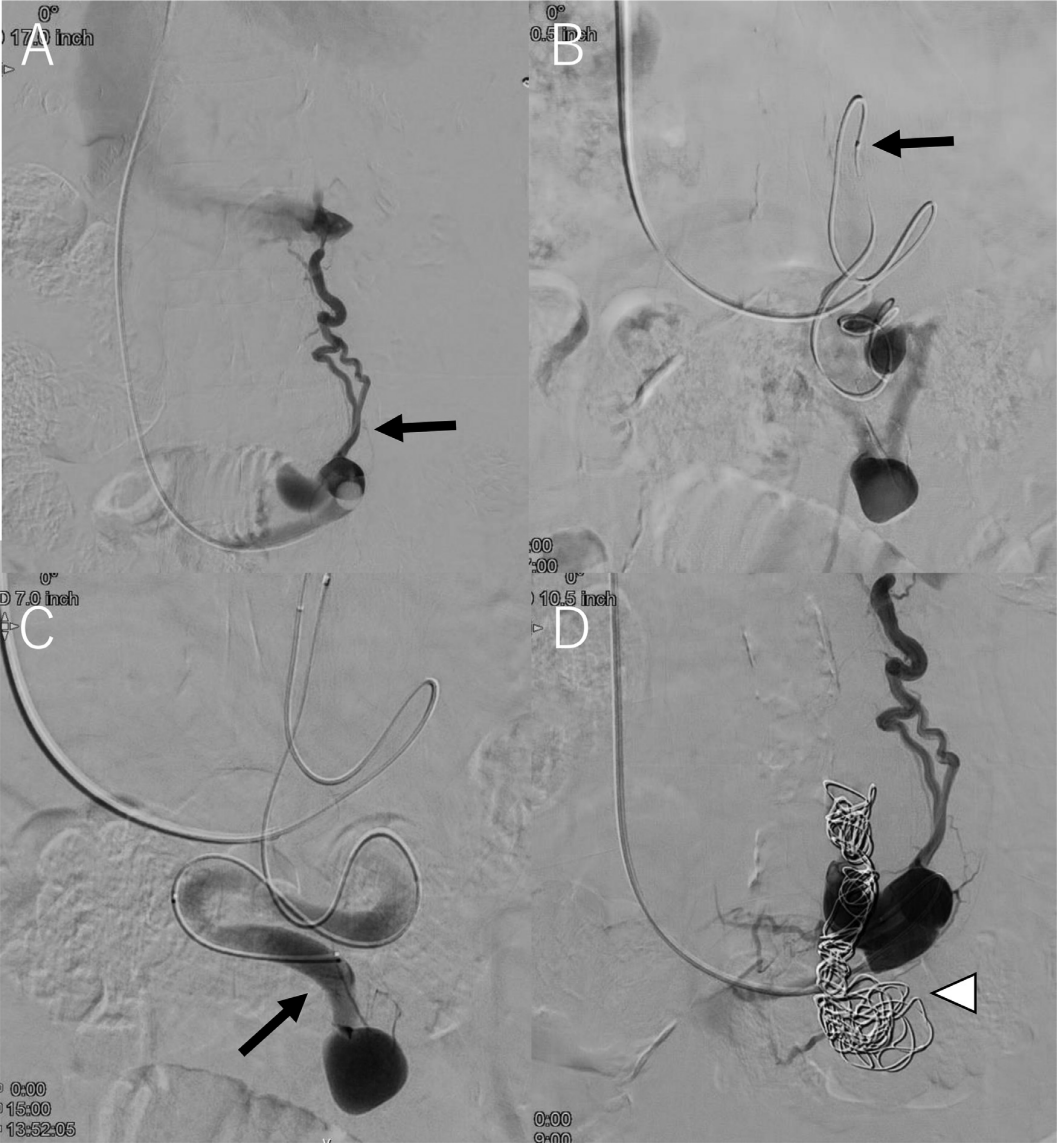
Retrograde shunt venogram during RTO with an antecubital vein approach using a steerable triaxial system. (A) Retrograde shunt venogram shows communicating route (black arrow) to the left renal vein. (B) Black arrow indicates the tip of the steerable microcatheter that is bent caudally at the inverted U curve of the shunt. (C) Black arrow indicates the tip of the small microcatheter that is located at the target point of the shunt. (D) White arrowhead indicates embolized microcoils.

**Fig. 6:**
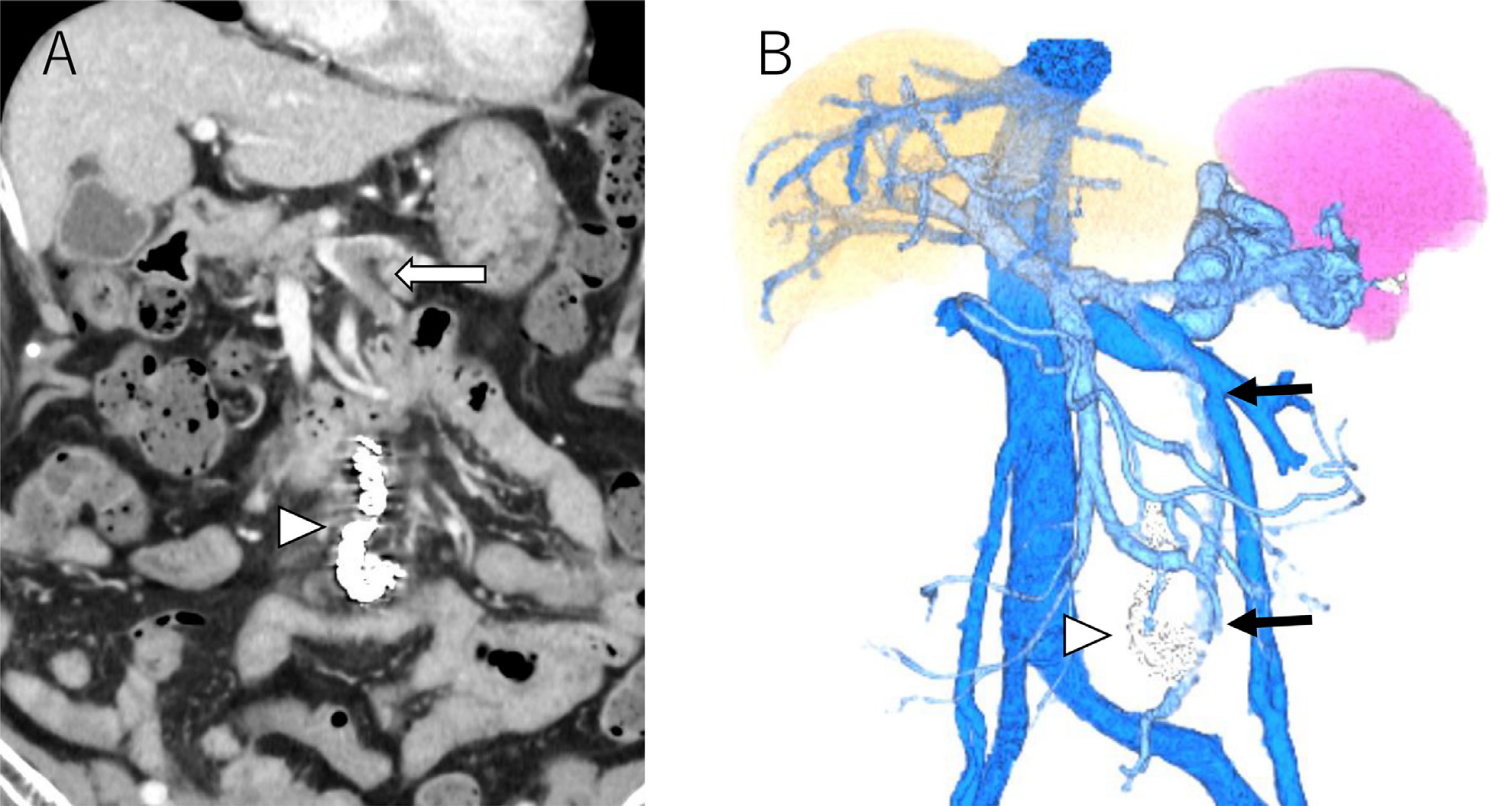
Contrast-enhanced CT and 3D-CT reconstruction images after treatment. (A) Coronal view of Contrast-enhanced CT shows the thrombus (white arrow) in the IMV and the splenic vein. White arrowhead indicates embolized microcoils. (B) 3D-CT reconstruction image after PSE and RTO shows the thrombus (black arrow) in the IMV. White arrowhead indicates embolized microcoils.

Two months after PSE, RTO with an antecubital vein approach using a steerable triaxial system was performed. The procedure was performed via the right antecubital vein using a 5-Fr. 25 cm long sheath (Radifocus introducer, Terumo, Tokyo, Japan). A 100 cm long 5-Fr. balloon catheter (Selecon MP cathterⅡ, Terumo, Tokyo, Japan), 130 cm long high-flow steerable microcatheter (2.9-Fr. distal, 2.9-Fr. proximal microcatheter; Leonis Mova, Sumitomo, Tokyo, Japan), and 160 cm long small microcatheter (1.9-Fr. distal, 1.9-Fr. proximal microcatheter, MARVEL, Tokai Medical Products, Kasugai, Japan), and 0.014-inch microguidewire (BEGIN; ASAHI INTEC, Nagoya, Japan) were used according to the previously reported steerable triaxial system [12], [13], [14]. First, a 5-Fr. balloon catheter was inserted into the outflow side of the IMV-iliac vein shunt, and the balloon with a diameter of 9 mm was inflated (Fig. 5A). Then, the high-flow steerable microcatheter, with the small microcatheter and a 0.014-inch microguidewire was advanced into the shunt. The tip of the steerable microcatheter was bent caudally at the inverted U curve of the shunt (Fig. 5B). The microguidewire was successfully inserted into the IMV side of the shunt. The small microcatheter was advanced to the target site with good support from the steerable microcatheter and balloon catheter (Fig. 5C). Then, embolization was performed using 0.014-inch microcoils (Target XL detachable coils, Stryker, Fremont, CA) (Fig. 5D). After the procedure, HVPG changed to 12.5 mm Hg. We intentionally preserved the splenorenal shunt to avoid a significant and sudden increase in portal venous pressure. The patient's postoperative course was uneventful. Three days after RTO, contrast-enhanced CT, and 3D-CT reconstruction images demonstrated the formation of thrombus in the IMV-left common iliac vein shunt (Fig. 6A and B), the plasma ammonia level reduced to 82 µg/dL. The patient's condition improved and she was discharged. Additional obliteration of the splenorenal shunt will depend on the course of ammonia levels in the future.

## Discussion

We reported a case of chronic portosystemic encephalopathy treated by an antecubital vein approach of RTO using a steerable triaxial system. There are 2 important issues in this case: 1. What is the best treatment strategy for chronic portosystemic encephalopathy? 2. What is an indication for an antecubital vein approach of RTO using a steerable triaxial system?

Encephalopathy caused by portosystemic shunt was termed “portosystemic encephalopathy” by Sherlock et al [1]. It occurs in compensated states of liver function. The presence of a portosystemic shunt contributes to portal decompression, however, it often causes hyperammonemia, hyperdynamic status, and narrowed arteriovenous oxygen content difference [8]. Management protocol for patients with cirrhosis and mental disturbance as proposed by Cordoba [16] proposed included: *1. Exclusion of neurological disorders, 2. A search of precipitating factors, 3. Evaluation of liver function and portal-systemic circulation.* According to this protocol, we can diagnose portosystemic encephalopathy. The pros and cons of shunt obliteration are comprehensively judged based on liver function, HVPG, degree of ascites, liver atrophy, and splenomegaly. Since shunt obliteration causes exacerbation of varices [17], its indication should be determined carefully. Significant and sudden increases in HVPG by shunt obliteration should be avoided. Therefore, when HVPG is 12 mm Hg or higher, partial or stepwise obliteration of shunts and combined use of PSE should be considered [18], [19], [20]. In this case, PSE was performed before RTO to control portal venous pressure. We also preserved the splenorenal shunt to buffer the RTO-induced portal pressure increase.

The jugular vein approach is more advantageous than the femoral vein approach for super-selective cannulation and HVPG evaluation because of the branching angle of the hepatic vein. The jugular vein approach has been our first choice until now [4,9]. However, carotid artery puncture, cervical hematoma, arteriovenous fistula, and neurological damage have been reported as complications of internal jugular vein puncture [21,22]. Although the cubital vein access route is long and the operability of the catheter could be inferior, this approach is easy to stop bleeding at the puncture site.

To achieve partial or stepwise obliteration of portosystemic shunts, the procedure should be easy to repeat and minimally invasive. The antecubital vein approach is one of the minimally invasive procedures. In hepatic venous catheterization, the antecubital vein approach has been performed as a minimally invasive procedure [23,24]. A 100 cm long 5-Fr. balloon catheter has been used for hepatic venous catheterization with the antecubital vein approach. We also used the same catheter and applied it to portosystemic shunt obliteration. Selective catheterization for portosystemic shunts is more difficult because these shunts are weaker and more fragile than the arteries. Because of these difficulties, we adopted a steerable triaxial system. The steerable triaxial system has been applied to arterial embolization such as arterial hemorrhage, aneurysm, and chemoembolization of hepatocellular carcinoma [12], [13], [14]. It was found that this system can also be applied to portosystemic shunt obliteration.

For this procedure to be successful, it is necessary to be able to perform antecubital vein puncture. In this procedure, using microcoils rather than liquid sclerosing agents is the most important method of embolization. We anticipate that this technique will play an important role in the treatment of portal hypertension in the future.

## Patient consent

Written informed consent was obtained from the patient for publication of this case report and accompanying images.
